# Pericyte Insulin Receptors Modulate Retinal Vascular Remodeling and Endothelial Angiopoietin Signaling

**DOI:** 10.1210/endocr/bqab182

**Published:** 2021-08-30

**Authors:** Nele Warmke, Fiona Platt, Alexander F Bruns, Claire H Ozber, Natalie J Haywood, Yilizila Abudushalamu, Charles Slater, Victoria Palin, Piruthivi Sukumar, Stephen B Wheatcroft, Nadira Y Yuldasheva, Mark T Kearney, Kathryn J Griffin, Richard M Cubbon

**Affiliations:** 1 Leeds Institute of Cardiovascular and Metabolic Medicine, University of Leeds, Leeds, UK; 2 Max-Delbrück-Centrum für Molekulare Medizin (MDC), Berlin, Germany

**Keywords:** Pericyte, endothelial, angiogenesis, venous, insulin, angiopoietin

## Abstract

Pericytes regulate vascular development, stability, and quiescence; their dysfunction contributes to diabetic retinopathy. To explore the role of insulin receptors in pericyte biology, we created pericyte insulin receptor knockout mice (PIRKO) by crossing PDGFRβ-Cre mice with insulin receptor (*Insr*) floxed mice. Their neonatal retinal vasculature exhibited perivenous hypervascularity with venular dilatation, plus increased angiogenic sprouting in superficial and deep layers. Pericyte coverage of capillaries was unaltered in perivenous and periarterial plexi, and no differences in vascular regression or endothelial proliferation were apparent. Isolated brain pericytes from PIRKO had decreased angiopoietin-1 mRNA, whereas retinal and lung angiopoietin-2 mRNA was increased. Endothelial phospho-Tie2 staining was diminished and FoxO1 was more frequently nuclear localized in the perivenous plexus of PIRKO, in keeping with reduced angiopoietin-Tie2 signaling. Silencing of *Insr* in human brain pericytes led to reduced insulin-stimulated angiopoietin-1 secretion, and conditioned media from these cells was less able to induce Tie2 phosphorylation in human endothelial cells. Hence, insulin signaling in pericytes promotes angiopoietin-1 secretion and endothelial Tie2 signaling and perturbation of this leads to excessive vascular sprouting and venous plexus abnormalities. This phenotype mimics elements of diabetic retinopathy, and future work should evaluate pericyte insulin signaling in this disease.

Pericytes are an integral component of the microvasculature, and are required for the stabilization and quiescence of the capillary endothelial cells they interact with (1). Newly forming angiogenic sprouts attract pericytes by secreting growth factors, such as platelet derived growth factor-β (PDGF-B) (2). Their presence prevents endothelial hyperproliferation and sprouting in growth factor–abundant environments, but also facilitates endothelial survival as growth factor abundance subsides, impacting on many facets of vascular biology (1, 2). They achieve this by closely wrapping around capillary endothelial cells, influencing their biology via direct interactions and paracrine signaling (2). The critical role of pericytes is exemplified by the embryonic lethality of PDGF-B knockout mice (3), which lose >95% of pericytes and exhibit highly abnormal vascular development and hemorrhage. Recent data from Pdgfb^ret/ret^ mice, which lose 70% to 80% of pericytes, reveal excessive angiogenic sprouting and a venous-shifted phenotype of endothelial cells, together with excessive endothelial expression of angiopoietin-2 (ANGPT2), a factor involved in angiogenic sprouting and vascular patterning (4). Attrition of pericytes occurs early in the natural history of diabetic microvascular disease, and is most widely appreciated in the retina, where it promotes features including venous dilation, hemorrhage, and excessive angiogenesis (5-7). Vascular endothelial insulin resistance is a key feature of diabetic vascular disease and is an important contributor to endothelial dysfunction (8). Notably, insulin receptor expression is comparable in pericytes and microvascular endothelial cells (9), and in vitro studies have shown greater proliferative responses to insulin in pericytes vs endothelial cells (10). However, the role of pericyte insulin signaling in vivo remains entirely unexplored and we set out to understand this in the context of developmental angiogenesis.

## Research Design and Methods

### Mouse Models and Husbandry

In order to knockout the insulin receptor in pericytes, mice with loxP sites flanking exon 4 of the *Insr* gene (referred to as floxed insulin receptor) (11) were crossed with mice expressing Cre-recombinase under control of the *Pdgfrb* promoter (referred to as Pdgfrb-Cre mice) (12). Their progeny were crossed to generate mice with homozygous deletion of the insulin receptor in Pdgfrb-expressing cells (ie, Pdgfrb-Cre.Insr^lox/lox^, referred to as PIRKO mice); these were always compared with Cre-negative Insr^lox/lox^ littermate controls. In order to define the spatial activity of Pdgfrb-Cre, these mice were separately crossed with mTmG reporter mice (13), in which the presence of active Cre modifies the mTmG reporter transgene from expression of membrane-targeted tandem Tomato to enhanced green fluorescent protein (eGFP). Founding insulin receptor floxed mice (strain 6955) and mTmG mice (strain 7576) were purchased from Jackson Laboratories (Farmington, CT, USA), while Pdgfrb-Cre mice were purchased from Taconic Biosciences (Germantown, NY, USA). All experimental mice were kept in a conventional animal facility with a 12-hour light/dark cycle and received a standard chow diet. Genotyping was performed by Transnetyx Inc. (Cordova, TN, USA) using real-time polymerase chain reaction (PCR) of ear notch genomic DNA. All procedures were approved by the Animal Welfare and Ethical Review Committee at the University of Leeds and were conducted in accordance with The Animals (Scientific Procedures) Act of 1986 Amendment Regulations 2012 (SI 2012/3039) under United Kingdom Home Office project licenses PL40/3523 and P144DD0D6.

### Assessment of Retinal Vasculature

Retinal angiogenesis was studied at P5, P10, and P15. For each experiment, mice from 3 litters were used to account for intrauterine and interindividual variability. Eyes were dissected and placed in 4% paraformaldehyde; retinas were then dissected, permeabilized, blocked and stained according to the method of Pitulescu et al. (14). Immunostaining was performed using primary antibodies against NG2 (15), Collagen IV (16), FoxO1 (17), DLL4 (18), EphB4 (19), COUP-TFII (20), and phosphoTie2 (21), and secondary antibodies against rabbit (Alexa Fluor 647) (22) or goat (Alexa Fluor 568) (23). Proliferation by EdU-incorporation was detected with a Click-iT EdU Alexa Fluor 647 Imaging Kit (Invitrogen C10340). Retinal vasculature was labeled with isolectin B4 conjugated to Alexa Fluor 488 or 647, and nuclei were labeled with Hoechst 33342. Retinas were flat mounted with ProLong Gold Antifade Mountant (Invitrogen P36930) and imaged using confocal microscopy (Zeiss LSM880) using FIJI (NIH, Bethesda, MD) for all analyses.

**Figure 1.**
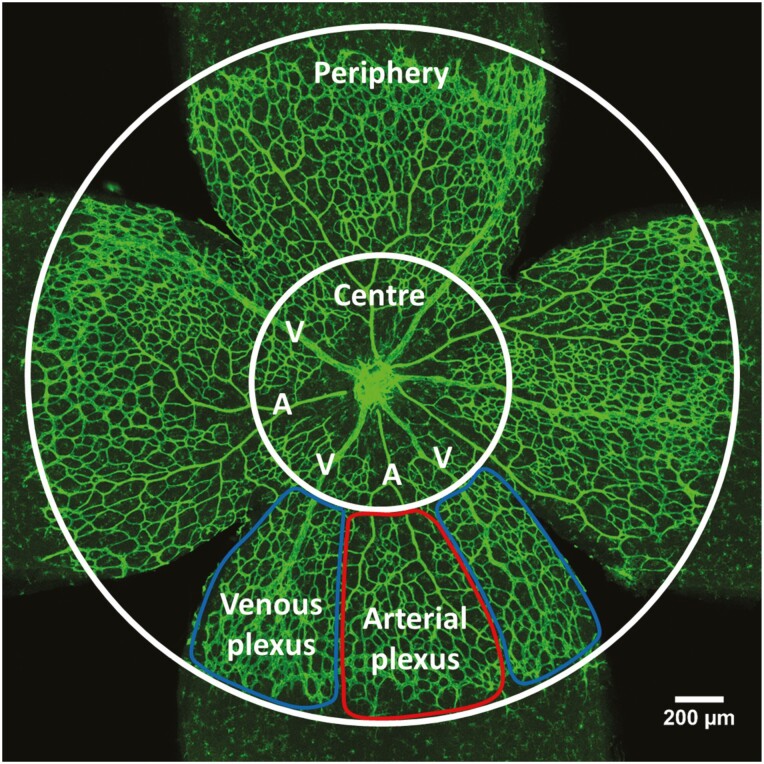
Retinal analysis regions of interest. P5 retinas were divided into center and periphery. The peripheral plexus can be divided into an arterial plexus around arteries (A) and a venous plexus around veins (V). Between arteries and veins stretches the capillary plexus.

For retinal radial outgrowth, vascularity, and branchpoint analysis, whole-retina tile-scan images were collected at 10× magnification using z-stacking and maximum intensity projection. Radial outgrowth was measured as distance between the optic disk and the angiogenic front. For vascularity and number of branchpoints, the vascular plexus was divided into central and peripheral zones bounded at one-third of the distance between the optic disk and the angiogenic front; in some analyses, the periphery was subdivided into arterial and venous regions (Fig. 1). Vascularity of the deeper plexus in P10 retinas was quantified as ratio of vascularized area of the deeper plexus and vascularized area of the fully outgrown superficial plexus. Vascular plexus branchpoints were defined in 5 to 8 (200 μm)^2^regions of interest in each retina, located between arteries and veins. Sprouting angiogenesis by active tip cells was imaged at 40× magnification in 6 to 8 regions at the vascular front between arteries and veins using z-stacking and maximum intensity projection; the number of sprouts was quantified per mm of vascular front.

Pericyte coverage by NG2 staining was imaged at 20× magnification and coverage was determined by thresholding pericyte NG2 signal over vascular isolectin B4 signal. In each retina 2 or 3 images of regions containing artery, vein, and capillary plexus were collected using tile-scanning, z-stacking, and maximum intensity projection. For quantifying vessel regression and proliferation by 5-ethynyl-2’-deoxyuridine (EdU) incorporation, 3 or 4 retina segments were imaged at 20× magnification, using tile scanning. Empty collagen IV, sleeves were identified as collagen IV structures between established vessels, but without overlaid isolectin B4 staining. The total number of EdU-positive nuclei were counted per segment. The number of regressed vessels or proliferating nuclei was then normalized per mm^2^ vascular area. FoxO1 nuclear localization was assessed by FoxO1/Hoechst colocalization inside the isolectin B4–stained vascular plexus. Three or 4 images of the arterial and venous regions were collected at 40× magnification, using z-stacking and maximum intensity projection. The number of vascular FoxO1-positive nuclei over all vascular nuclei was analyzed in a (150 μm)^2^ region of interest adjacent to an artery or a vein. Tie2 activation was analyzed by mean staining intensity in artery, arterial plexus, venous plexus, and vein, and corrected for mean staining intensity in artery. In each retina, 2 or 3 images were collected at 40× magnification. Images had to contain an artery, vein, and the capillary plexus using tile-scanning, z-stacking, and maximum intensity projection. High background staining required processing of the images by removing bright outliers (antibody precipitates) and background subtraction. The isolectin B4 signal was used to create a mask and to remove any Tie2 signal from outside the vasculature. Mean staining intensity was measured across an individual vessel and 3 measurements were taken per region.

### Isolation and Culture of Murine Pericytes

Pericytes were isolated from the brains of adult mice (8-10 weeks of age), adapting the method of Tigges et al. (24). Brain tissue, collected after cervical dislocation, was manually minced and digested in 1 mg/mL collagenase/dispase (Roche 11097113001) on a MACSmix Tube Rotator (Miltenyi Biotec) at 37°C for 45 to 60 minutes. Myelin was removed by centrifugation after resuspension of the brain pellet in Dulbecco’s modified Eagle’s medium with 20% bovine serum albumin (BSA). Cell pellets were then cultured at 37°C in 5% CO_2_ on 2% gelatin-coated plates using Endothelial Cell Growth Medium MV2 (PromoCell C-22022, supplemented with an additional 10% fetal bovine serum and 1% antibiotic–antimycotic solution (Sigma-Aldrich A5955)). Upon confluence, cells were trypsinized with 0.25% Trypsin-EDTA (Thermofisher 2520056) and transferred into a new culture vessel with subsequent culture in Pericyte Medium (ScienCell 1201) until passage 5. To confirm the phenotype with immunostaining, cells were fixed in 4% paraformaldehyde, and permeabilized and blocked in 0.25% PBS-Triton-X100 supplemented with 1% BSA and 5% goat serum. Cells were incubated overnight in antibodies against NG2 (15), or VEGFR2 as an endothelial marker (25). Cells were then incubated with a fluorescent antirabbit secondary antibody (22), followed by nuclear counterstaining with Hoechst 33342. Confocal microscopy was performed (Zeiss LSM880) at 40× magnification.

### Culture of Human Pericytes and Endothelial Cells

Commercially available human brain pericytes (HBPCs) were purchased from ScienCell and cultured in pericyte medium (ScienCell 1200 and 1201). Experiments were performed at passage 4 to 8. HBPCs were transduced using short hairpin RNA (shRNA) lentivirus particles, at a concentration of 15 multiplicity of infection, against the insulin receptor (Sigma-Aldrich SHCLNV-NM000208/ TRCN0000196786) or against green-fluorescent protein (Sigma-Aldrich SHC002H) to create shIR and control HBPCs, respectively. To assess ANGPT1 secretion in shIR and control HBPCs, conditioning was performed for 24 hours in M199 basal media (Sigma-Aldrich M4530) supplemented with 2% fetal bovine serum, 1% pericyte growth supplement (ScienCell 1252), and 100 nM insulin. ANGPT1 concentration in human pericyte conditioned medium was quantified using the Quantikine Human Angiopoietin-1 ELISA (26). Conditioned medium from shIR and control HBPCs was also applied to human umbilical vein endothelial cells (HUVECs; PromoCell 12200) to assess Tie2 activation. HUVECs were serum-deprived in M199 basal media for 2 hours and conditioned medium was applied for 15 minutes before collecting cell lysates for immunoblotting.

### Quantitative Polymerase Chain Reaction

Total RNA was isolated using TRI Reagent Solution (Thermo Fisher Scientific). For tissue RNA extraction, tissue was homogenized in TRI Reagent using a Qiagen TissueLyser at 25 Hz. Phase separation was performed with 1-bromo-3-chloropropane and the RNA-containing upper phase was collected. By adding isopropanol, RNA was precipitated after centrifugation and the RNA pellet was washed with 99.5% ethanol. RNA was dissolved in DNase/RNase free water and quantified by using a DeNovix DS-11 FX + spectrophotometer. Reverse transcription was performed using the High-Capacity RNA-to-cDNA Kit (Thermo Fisher Scientific 4387406). For quantitative PCR, PrecisionPLUS qPCR Master Mix (Primer Design PPLUS-CL) and the following 6-carboxyfluorescein (FAM) labeled TaqMan probes were used: mouse *insr* (Mm00439688_m1); mouse *actb* (Mm02619580_g1); mouse *angpt1* (Mm00456503_m1); mouse angpt2 (Mm00545822_m1), mouse *hes1* (Mm01342805_m1), mouse *hey1* (Mm00468865_m1), and mouse *nrarp* (Mm00482529_s1). Amplification of gene fragments was assessed using a Roche LightCycler 480.

### Immunoblotting

Protein concentrations in cell lysates were quantified using the Pierce BCA Protein Assay Kit (Thermo Fisher Scientific). Protein was separated by denaturing gel electrophoresis on NuPAGE 4-12% Bis-Tris Protein Gels using NuPAGE MES SDS running buffer. Protein was transferred onto PVDF membranes using a Trans-Blot Turbo Transfer System. Membranes were blocked in blocking buffer (tris-buffered saline with 0.1% Tween-20 (TBS-T 0.1%, 50 mM Tris, 150 mM NaCl at pH 7.6, 0.1% Tween-20 supplemented with 5% BSA in PBS) before overnight incubation in primary antibodies against HSP90 (27), beta-actin (28), phosphoTie2 (21), or insulin receptor beta subunit (29), diluted in blocking buffer at 4°C. Washes in 0.02% TBS-T were performed before incubation with a secondary antibody against mouse (30), or rabbit (31), diluted in blocking buffer or 0.02% TBS-T for 2 hours at room temperature. Membranes were imaged with a Syngene G:BOX after incubation with Immobilon Western Chemiluminescent HRP Substrate (Merck Millipore).

### Statistics and Measures to Reduce Measurement Bias

Statistical analysis was performed using GraphPad Prism 7. Data are presented as mean ± standard error of the mean (SEM) and results are considered significant when *P* ≤ .05. Where appropriate, an unpaired or paired t-test has been performed; when distributions were heterogeneous in variability, a Welch-t-test was performed instead. Analysis of 2 independent factors on 1 dependent variable was performed by a 2-way analysis of variance (ANOVA). In order to reduce measurement bias, all experiments were performed and analyzed by investigators blinded to genotype or treatment allocation until experiments were completed.

## Results

### PIRKO Exhibit Retinal Venous Plexus Hypervascularity

**Figure 2.**
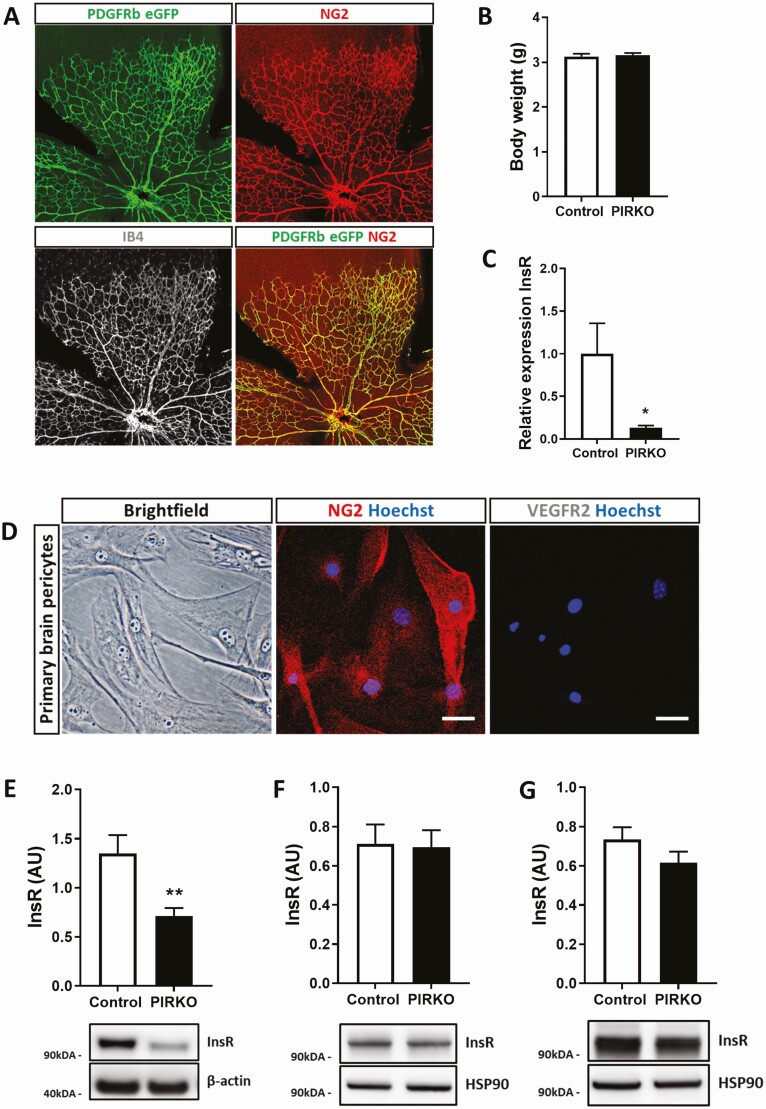
Confirmation of appropriate pericyte insulin receptor knockout. (A) Cre-recombination was assessed in PDGFRβ-Cre^mTmG+/–^ mice. Endogenous eGFP expression colocalizes with pericyte NG2 expression in P5 retinas. The vasculature was labeled with isolectin B4 (gray). (B) There is no difference in body weight at P5 between PIRKO and control. (C) Brain pericytes were isolated from adult control and PIRKO mice. Knockdown of the insulin receptor was confirmed on RNA level (relative to β-actin). (D) Isolated brain pericytes at passage 5 express the pericyte marker NG2 (central panel), but not the endothelial marker VEGFR2 (right panel), scale bar 50 µm. (E) Insulin receptor protein level in reduced in isolated brain pericytes from PIRKO. (F) PDGFRβ-targeted insulin receptor knockdown does not affect whole tissue insulin receptor expression in P5 retinas and (G) P5 lungs. Data presented as mean ± SEM, unpaired t-test, *P < .05, **P < .01, n = 6, 6 (C), n = 9, 9 (E), n = 7, 7 (F,G).

**Figure 3.**
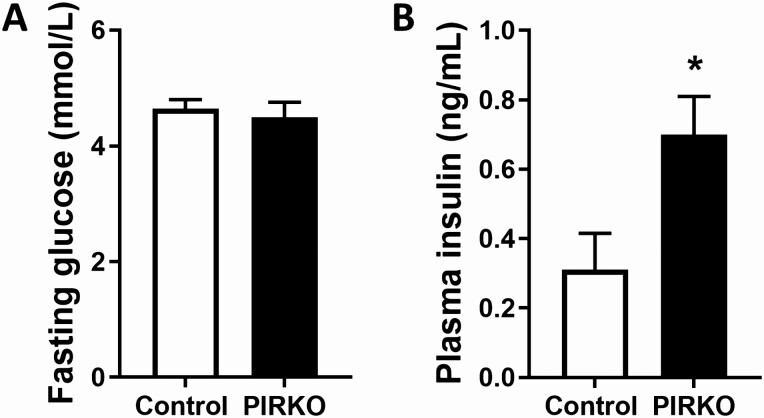
Fasting serum glucose and insulin concentrations. Eight-week-old PIRKO mice exhibit similar fasting serum glucose concentrations (A), but increased fasting serum insulin concentrations (B), compared with wildtype littermates. Data presented as mean ± SEM, unpaired t-test, *P < .05, n = 12, 13 (A, B).

In order to define the spatial activity of Pdgfrb-Cre, these mice were crossed with mTmG reporter mice (13), in which the presence of active Cre modifies the mTmG reporter transgene from expression of membrane-targeted tandem Tomato to eGFP. Examination of the P5 retina confirmed appropriate vascular development, with eGFP expression colocalizing only with immunostaining for the mural cell marker NG2 (Fig. 2A). Next, Pdgfrb-Cre mice were crossed with insulin receptor floxed mice to generate mice with homozygous deletion of the insulin receptor in Pdgfrb expressing cells (now referred to as PIRKO); these were always compared with Cre-negative Insr^lox/lox^ littermate controls. PIRKO were born at expected frequencies, exhibited no gross developmental abnormalities and had comparable weight to littermate controls (Fig. 2B). Isolation and culture of brain pericytes from PIRKO revealed these to express appropriate pericyte markers and exhibit approximately 90% reduction in *Insr* mRNA (Fig. 2C and 2D); moreover, these had a 50% reduction in insulin receptor protein, with apparent truncation of expressed protein based on reduced molecular mass (Fig. 2E). Notably, insulin receptor protein was not reduced in whole retinal or lung lysates from P5 PIRKO mice (Fig. 2F and 2G). In 8-week-old mice, fasting serum glucose was comparable in PIRKO mice and wildtype littermates, although fasting serum insulin was greater in PIRKO (Fig. 3A and 3B).

**Figure 4.**
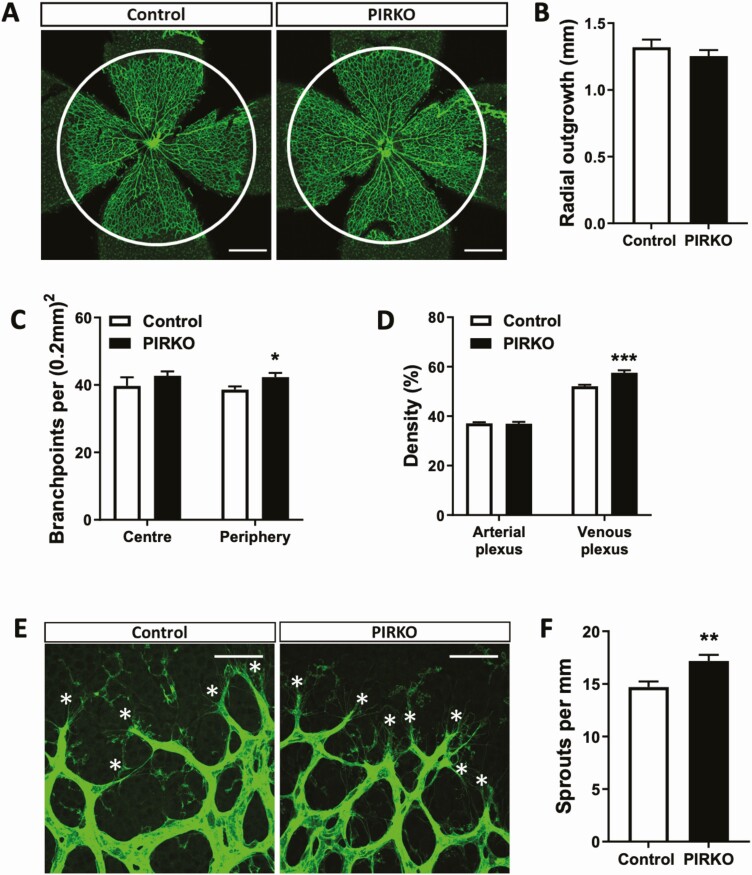
Perivenous density and sprouting is increased in PIRKO. (A) Whole-mounted retinas at P5 were stained with isolectin B4 to assess developmental angiogenesis, scale bar 500 µm. (B) Radial outgrowth from the optic disk is the same in both groups. (C) Number of branchpoints is increased in the peripheral retina in PIRKO, but unchanged in the center of the retina. (D) Density of the arterial plexus is not different between the groups, but density of the venous plexus is increased in PIRKO. (E) Vascular sprouting at the emerging front was assessed by isolectin B4 staining at P5. Tip cells are labeled with an asterisk, scale bar 50 µm. (F) Number of sprouts is increased in PIRKO. Data presented as mean ± SEM, unpaired t-test, *P < .05, **P < .01, ***P < .001, n = 9, 11 (B-D) and n = 8, 11 (F).

**Figure 5.**
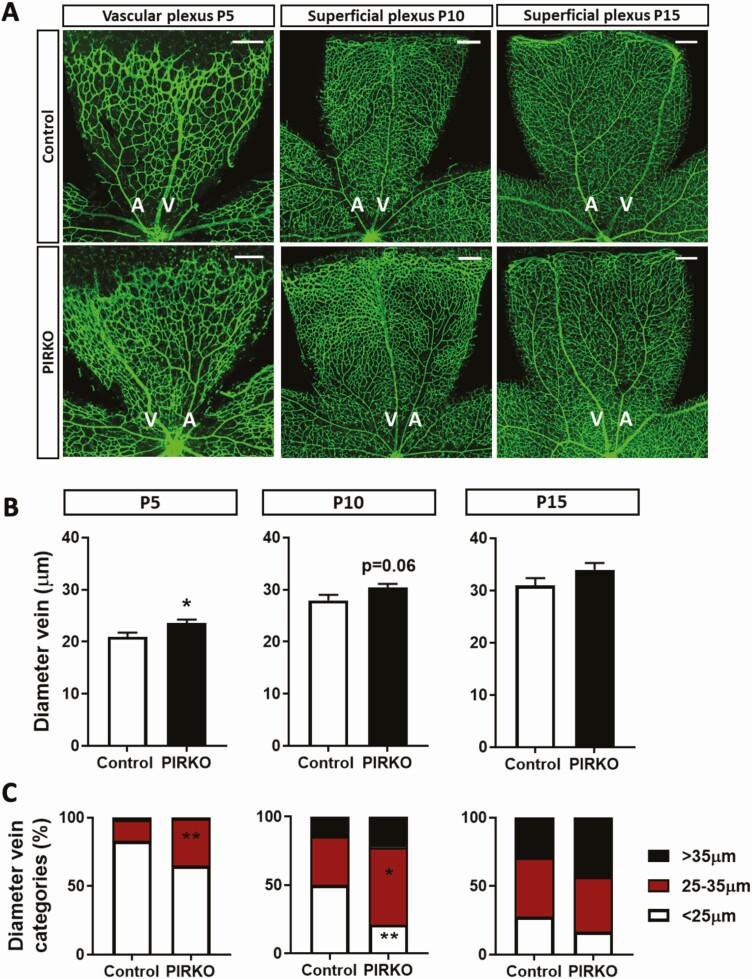
Vein diameter is increased in PIRKO. (A) Retinas at P5, P10 and P15 were whole-mounted and stained with isolectin B4 to assess vein diameter, scale bar 200 µm. (B) Vein diameter is increased in PIRKO at P5 and normalizes by P15. (C) Veins at P5, P10, and P15 were divided into 3 (2 at P5) equally long segments (center, middle, periphery) and proportion of segments <25 µm, between 25 and 35 µm, and >35 µm were determined. P5 retinas show an increase in larger segment vessel in PIRKO, which normalizes by P15. Data presented as mean ± SEM, unpaired t-test (B) or 2-way ANOVA with Sidak’s multiple comparison test (C), *P < .05, **P < .01, n = 9, 11 at P5, n = 7, 9 at P10, n = 7, 8 at P15; A, artery; V, vein.

**Figure 6.**
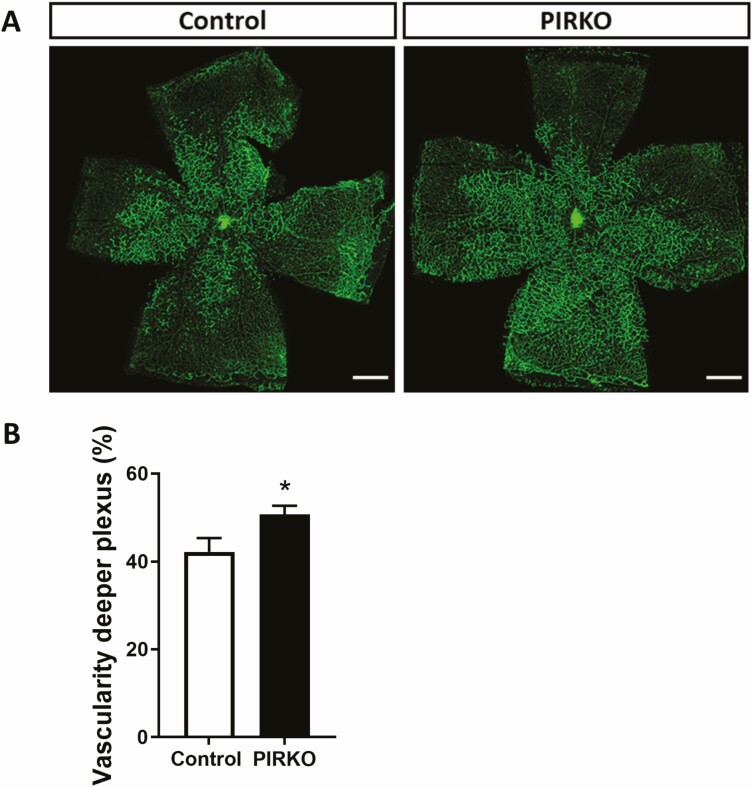
Deep retinal vascular plexus development. (A) At P10, retinas were stained with isolectin B4 to assess vascularity of the deeper plexus, scale bar 500 µm. (B) Vascularity of the deeper plexus is increased in PIRKO. Data presented as mean ± SEM unpaired t-test, *P < .05, n = 7, 9.

Retinal vascular development was defined by staining P5 retinal wholemounts with isolectin B4, which highlights vascular endothelium (Fig. 4A). Radial outgrowth of the emerging plexus was comparable in PIRKO and control littermates (Fig. 4B), although PIRKO exhibited increased branching complexity in the peripheral half of the plexus (Fig. 4C). We noted increased vascularity in the regions adjacent to retinal veins and quantified this as vascular density (the proportion of retinal area covered by vasculature) separately in the perivenous and arterial plexi. Although no difference was noted between PIRKO and control littermates in arterial plexus vascular density, this was clearly increased in the venous plexus of PIRKO (Fig. 4D). Using higher magnification images of the emerging vascular front (Fig. 4E), we also defined the number of sprouting tip cells, normalized to the perimeter of the vascular front, and found this to be increased in PIRKO (Fig. 4F). In order to better understand the retinal venous plexus abnormalities of PIRKO, we then conducted additional analyses at P5, P10, and P15 (Fig. 5A). These revealed that the perivenous hypervascularity diminished with time, although was still evident at P10. Beyond increased venous plexus area, we also noted increased retinal vein diameter in P5 and P10 PIRKO mice, with a nonsignificant increase evident at P15 (Fig. 5B and 5C). At P10, we also studied the deep vascular plexus, which sprouts from the primordial superficial layer, and found significantly increased vascular area in PIRKO mice (Fig. 6A and 6B). In summary, these data suggest PIRKO have increased vascular sprouting and abnormal venous plexus development in early postnatal life.

**Figure 7.**
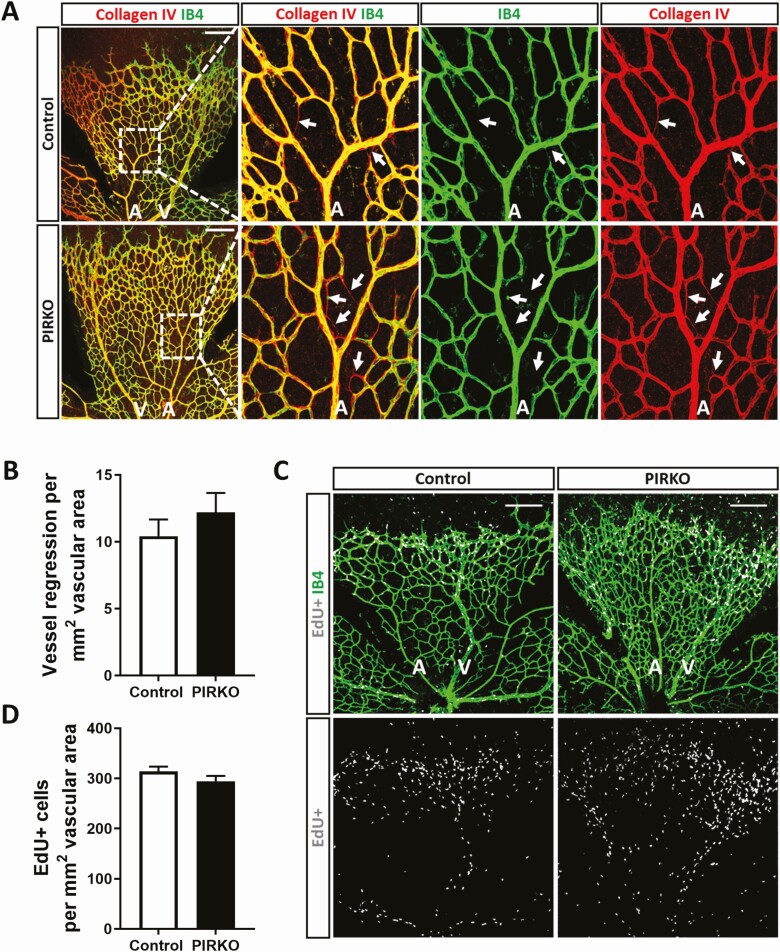
Assessment of vascular regression and endothelial proliferation. (A) Vessel regression at P5 was assessed by collagen IV empty sleeves, regressed vessels are labeled with an arrow, scale bar 200 µm. (B) Vessel regression is similar between PIRKO and control. (C) Vascular cell proliferation was assessed by EdU-incorporation, scale bar 200 µm. (D) There is no difference in vascular cell proliferation between the groups. Data presented as mean ± SEM, unpaired t-test, not significant, n = 13,13 (B), n = 8,8 (D); A, artery; V, vein.

**Figure 8.**
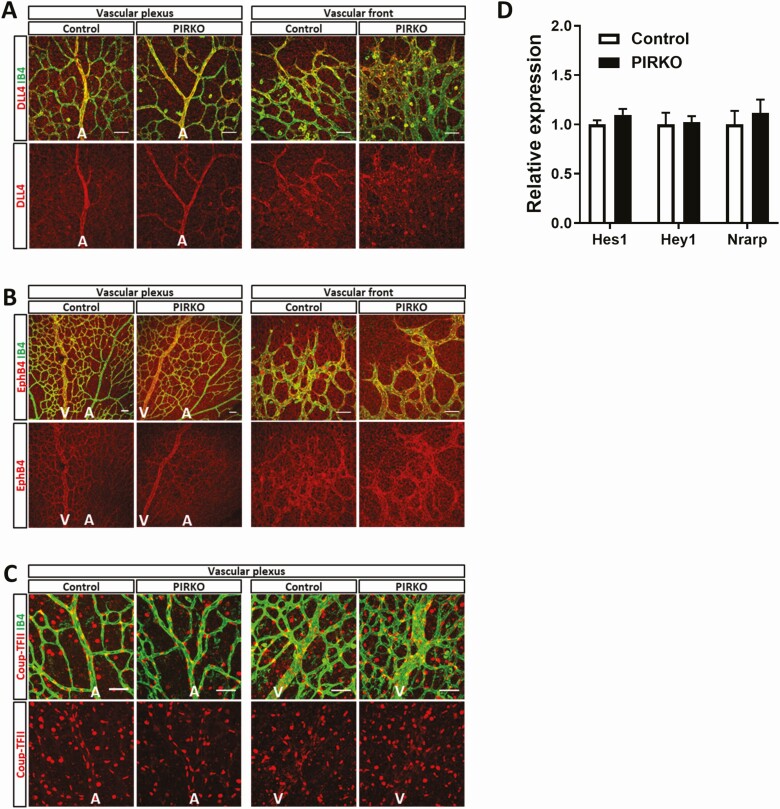
Retinal arteriovenous commitment marker expression. Arterial- and venous commitment markers (A) DLL4, (B) EphB4, and (C) Coup-TFII were qualitatively assessed in P5 retina and appear to be adequately expressed at the vascular plexus and vascular front, scale bar 50 µm. (D) mRNA expression of Notch target genes Hes1, Hey1, Nrarp are unchanged in PIRKO in P5 retinas. Data (D) are presented as mean ± SEM, relative expression corrected for β-actin, unpaired t-test not significant, n = 6,9 (A-C), n = 7,5 (D); A, artery; V, vein.

**Figure 9.**
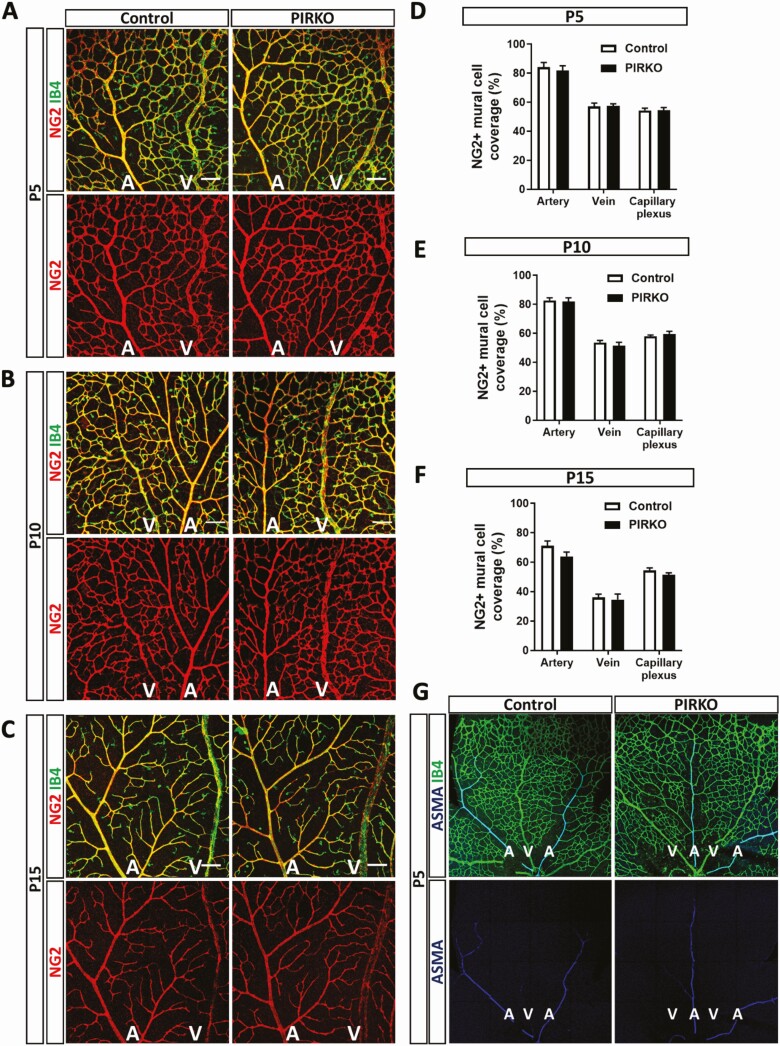
Mural cell coverage is not affected in PIRKO. Mural cell coverage was assessed by NG2 staining at (A) P5, (B) P10, and (C) P15, scale bar 100 µm. NG2 + mural cell coverage is unchanged in arteries, veins and the capillary plexus at (D) P5, (E) P10, and (F) P15. (G) P5 retinas were stained for α-smooth muscle actin (ASMA) to locate vascular smooth muscle cells (VSMCs). Arteries, but not the capillary plexus or veins are covered by ASMA-expressing VSMCs. Data presented as mean ± SEM, 2-way ANOVA with Sidak’s multiple comparison test, not significant, n = 9, 9 at P5, n = 8, 8 at P10, and n = 7, 8 at P15; A, artery; V, vein.

Since hypervascularity could be explained by diminished vascular regression (ie, pruning of unnecessary vessels, which is essential for normal vascular development), we stained retinas for collagen IV (Fig. 7A); empty collagen IV sleeves without overlying isolectin B4 staining provide evidence of recent vascular regression. We observed no difference in the number of these events per area of retinal vasculature (Fig. 7B). Excessive proliferation of endothelial cells could also contribute to hypervascularity and so we injected P5 pups with EdU, a nucleoside analogue that incorporates into newly forming DNA, allowing fluorescent labeling of proliferating cell nuclei (Fig. 7C). Again, we saw no difference in the number of EdU staining endothelial (isolectin B4 stained) nuclei, normalized to retinal vascular area, in PIRKO mice vs littermate controls (Fig. 7D). Another potential explanation for abnormal venous plexus development is abnormal arteriovenous patterning and so we stained retinas with Dll4 (enriched in arteries), EphB4, and Coup-TFII (both enriched in veins), yet these revealed appropriate staining patterns in PIRKO (Fig. 8A-8C). Since Notch signaling is implicated in vascular patterning and development, we also performed qPCR for canonical Notch targets (*Hes1*, *Hey1*, *Nrarp*) in whole retinal tissue, although no differences were observed (Fig. 8D). Given our focus on pericytes, we also stained retinas for NG2 in order to define mural cell coverage of the vasculature. The area of retinal arteries, veins and capillary plexi covered with NG2 staining was comparable in PIRKO mice, vs littermate controls, at P5, P10, and P15 (Fig. 9A-9F). Staining for the vascular smooth muscle marker alpha-smooth muscle actin also revealed no differences at P5, with coverage appropriately restricted to developing retinal arteries (Fig. 9G).

### Angiopoietin-1 Signaling Is Reduced in PIRKO

**Figure 10.**
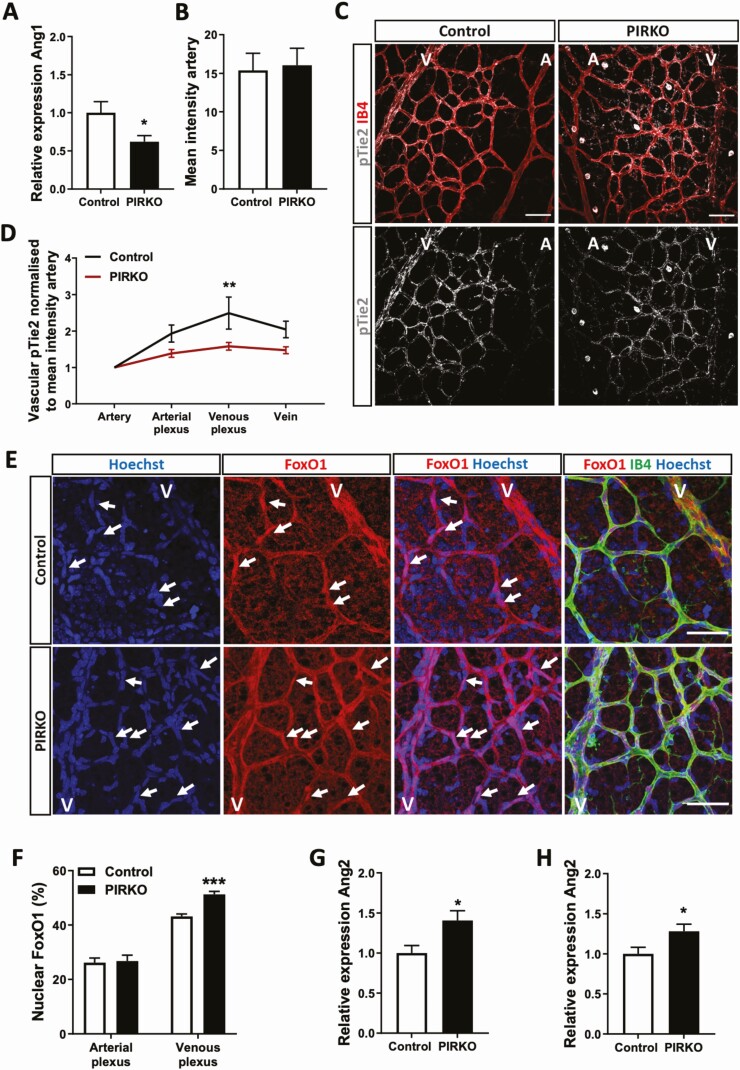
Angiopoietin/ Tie2 signaling is altered in PIRKO. (A) Angpt1 mRNA expression is reduced in isolated brain pericytes from PIRKO compared with control. (B) and (C) Phosphorylation of Tie2 was assessed in P5 retinal territories (C), and normalized to the mean staining intensity of pTie2 in arteries (B), scale bar 50 µm. (D) pTie2 staining is reduced in the venous plexus PIRKO. (E) P5 retinas were stained against FoxO1 and nuclear FoxO1 localization was assessed by FoxO1/Hoechst colocalization. FoxO1 positive nuclei are labeled with an arrow, scale bar 50 µm. (F) Nuclear FoxO1 in the arterial plexus is similar in both groups, whereas nuclear FoxO1 localization is increased in the venous plexus in PIRKO compared to control. (G) Angpt2 mRNA expression (relative to β-actin) is increased in P5 retinas and (H) P5 lungs in PIRKO compared to control. Data presented as mean ± SEM, unpaired t-test (A, B, F, G, and H), or 2-way ANOVA with Sidak’s multiple comparison test (D), *P < .05, ***P < .001, n = 6, 6 (A), n = 10, 10 (B, D) n = 7, 10 (F) n = 7, 7 (G, H); A, artery; V, vein.

The altered retinal endothelial biology of PIRKO mice led us to explore other known mechanisms of pericyte–endothelial communication that can influence venous development. Angiopoietins are a family of ligands that profoundly alter endothelial biology, acting via endothelial Tie2 receptors that signal downstream via Akt and the transcription factor FoxO1 (32). Tie2, Akt, and FoxO1 have all been implicated in venous development (33-36). Pericytes secrete Angiopoietin-1, an agonist of Tie2, encoded by the *Angpt1* gene. We observed that *Angpt1* mRNA was decreased in cultured brain pericytes from PIRKO (Fig. 10A). In order to explore the relevance of this in vivo, we then stained retinas for activated Tie2 receptors, using its phosphorylation at Y1102 as a proxy of activation. In spite of similar absolute staining intensity in retinal arteries (Fig. 10B), we noted a pronounced reduction in perivenous capillary plexus Tie2 phosphorylation, normalized to arterial signal in each image (Fig. 10C and 10D). Since downstream signaling via Akt to FoxO1 is thought to be important in Tie2-mediated venous patterning, we stained retinas for FoxO1 (Fig. 10E), taking advantage of the fact that diminished Tie2/Akt signaling should *increase* nuclear localization of FoxO1. In support of reduced perivenous Tie2 signaling, we found endothelial nuclear localization of FoxO1 was increased in perivenous regions of PIRKO, vs littermate controls, with no difference in periarterial regions (Fig. 10F). Importantly, endothelial FoxO1 activity induces expression of angiopoietin-2 (a context-specific antagonist of ANGPT1, expressed predominantly by endothelial cells, encoded by the gene *Angpt2*) and we observed increased *Angpt2* mRNA in PIRKO retinas and lung (Fig. 10G and 10H). Hence, PIRKO mice have reduced peri-venous Tie2 signaling, associated with reduced pericyte expression of its agonist ANGPT1, and increased expression of its antagonist ANGPT2; collectively, these would be expected to promote venous development.

### Insr Knockdown in Human Brain Pericytes Recapitulates Abnormal Angiopoietin Signaling

**Figure 11.**
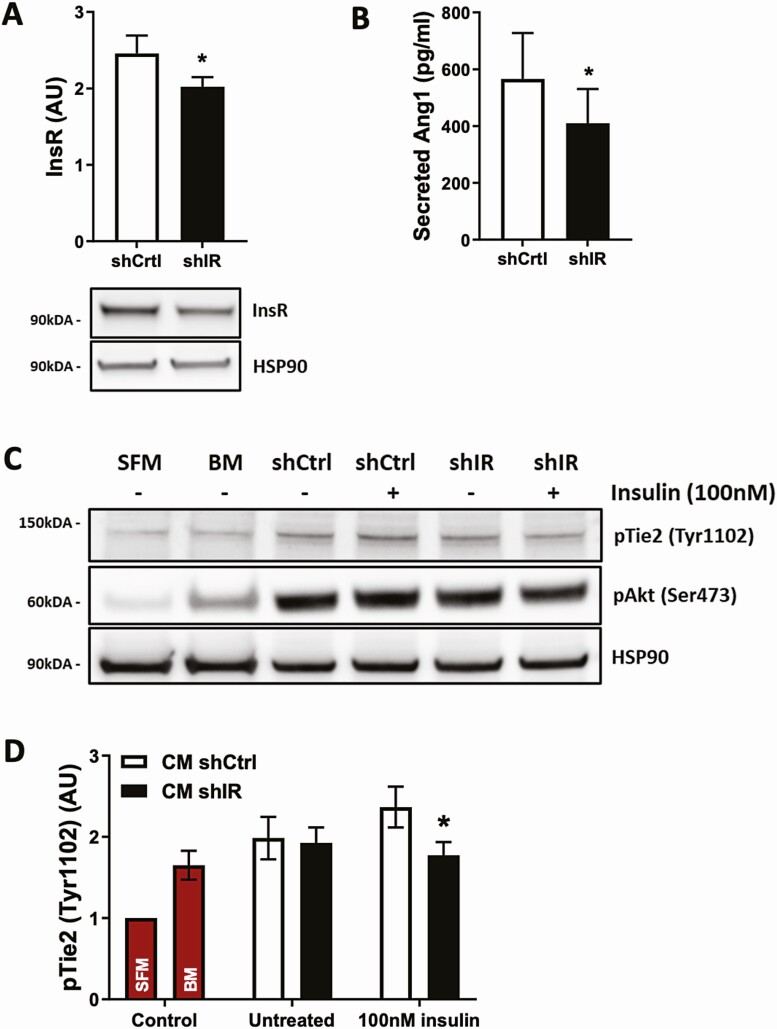
Pericyte conditioned medium activates the Tie2/Akt signaling axis in HUVECs. Knockdown in human brain pericytes was performed using short hairpin RNA transduction particles against the insulin receptor (shIR) or green-fluorescent protein (shCtrl). (A) Knockdown efficiency with a multiplicity of infection of 15 results in a minor reduction of insulin receptor expression on protein level in shIR. (B) Secretion of Angpt1 into cell culture supernatant is reduced in shIR compared to shCtrl after 24 hours, upon stimulation with 100 nM insulin. (C) HUVECs were treated with serum-free cell-starvation medium (SFM), basal medium (BM) used for conditioning, or shCtrl or shIR conditioned medium (CM), either collected under untreated or 100 nM insulin-stimulated conditions, for 15 minutes to assess activation of the Tie2/Akt signaling axis. (D) Tie2 activation in HUVECs treated with conditioned medium from 100nM insulin-stimulated shIR is reduced compared to shCtrl. Data presented as mean ± SEM, paired t-test (used because within each experimental replicate, shIR or shCtrl were transduced in pericytes derived from a single donor), *P < .05, n = 9, 9 (A, D) and n = 5, 5 (B).

**Figure 12.**
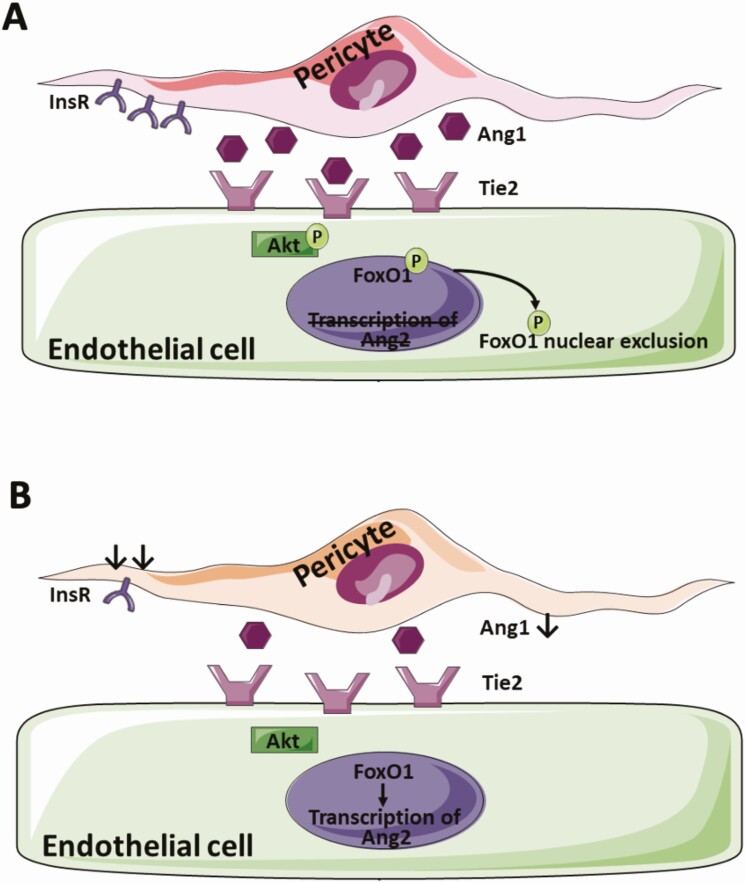
Proposed link between pericyte insulin signaling and endothelial cell biology. (A) In health, pericyte insulin signaling regulates Ang1 secretion and hence pericyte-endothelial crosstalk via Ang1-Tie2, promoting endothelial FoxO1 nuclear exclusion and preventing Ang2 transcription. (B) In PIRKO, insulin receptor signaling is disrupted, which reduces Ang1 secretion and downstream activation of endothelial Tie2. The transcription factor FoxO1 remains nuclear and allows transcription of Ang2.

Finally, we asked whether these data are relevant to human pericyte biology, and so knocked down the insulin receptor in human brain pericytes using lentiviral transduction of *Insr* shRNA (shIR) vs control GFP-targeting shRNA (shCtrl). This achieved approximately 25% reduction in insulin receptor protein (Fig. 11A). We then quantified secreted angiopoietin-1 protein in conditioned medium from these pericytes and found that *Insr* knockdown reduced ANGPT1 concentrations (Fig. 11B), in keeping with data from cultured PIRKO pericytes. To establish whether this was functionally relevant for venous endothelial cells, we then exposed HUVECs to conditioned medium for 15 minutes before lysing cells for phospho-Tie2 immunoblotting. This revealed that conditioned medium from *Insr* knockdown pericytes was significantly less able to activate Tie2 in HUVECs (Fig. 11C and 11D), supporting our hypothesis that insulin signaling in pericytes regulates pericyte to endothelial cell communication via endothelial Tie2 signaling (Fig. 12).

## Discussion

Our work provides the first ever in vivo assessment of pericyte insulin receptor function and points to its fundamental role in vascular quiescence and patterning. Specifically, we show that pericyte insulin receptors suppress vascular sprouting and influence venous plexus patterning during developmental angiogenesis, and link this with regulation of paracrine angiopoietin signaling. While we have not studied pathological angiogenesis, our data may be relevant to disease states associated with altered insulin receptor biology, such as diabetic retinopathy, and future work should address this possibility and its translational potential.

### Pericytes, Angiopoietins, and Vascular Biology

The essential role of pericytes during vascular development has already been alluded to, based upon the embryonic lethality and highly abnormal vascular development of PDGF-B knockout mice (3), which lose >95% of pericytes. Pdgfb^ret/ret^ mice, which lose 70% to 80% of pericytes, survive into adulthood and exhibit excessive angiogenic sprouting with venous-shifted phenotype of endothelial cells, together with excessive endothelial expression of ANGPT2 (4). Although their phenotype is more extreme and sustained than PIRKO, there are clear parallels in terms of vascular morphology and altered angiopoietin expression. However, we did not observe a reduction in pericyte coverage associated with these structural and molecular abnormalities, suggesting instead that pericyte function is altered. While this dysfunction is likely to encompass more than just reduced ANGPT1 secretion, we were able to elicit diminished paracrine ANGPT1 signaling in venous plexus endothelial cells, evidenced by: reduced Tie2 phosphorylation; increased nuclear FoxO1 localization; and increased *Angpt2* mRNA (likely resulting in further reduction in ANGPT1 signaling due to its antagonistic role). ANGPT1 secreted by pericytes signals via endothelial Tie2 receptors (37), resulting in potent activation of the kinase Akt, which in turn phosphorylates the transcription factor FoxO1 (32), directing it to a cytoplasmic location; among many other effects, this represses *Angpt2* expression (32). Importantly, diminished endothelial signaling at either Tie2, Akt, or FoxO1 axis is consistently associated with abnormal venous patterning (33-36). Hence, the venous plexus hypervascularity we observed is consistent with the molecular perturbations we describe. Moreover, the increased vascular sprouting of PIRKO is consistent with their increased expression of ANGPT2, given that this has an important role in sprouting angiogenesis (2, 38). It would be interesting to explore ANGPT2 secretion by human endothelial cells exposed to conditioned medium from insulin resistant pericytes and future studies should address this. However, it should be noted that angiopoietin signaling is complex, with Tie2 expression and angiopoietin responsiveness also being evident in pericytes (39, 40), and our findings should be considered in this context. Moreover, angiopoietins are part of a much broader angiogenic regulatory network, and it is particularly important to consider their interaction with vascular endothelial growth factor (VEGF), the principal angiogenesis stimulating growth factor. While we did not define VEGF bioactivity in the retina of PIRKO mice, the work of Park et al. illustrates that loss of pericytes is associated with a much more pronounced phenotype (mimicking diabetic retinopathy) in the presence of increased VEGF (41). Park and colleagues also confirm that endothelial FoxO1, but not pericyte-derived ANGPT1, contributes to the vascular dysfunction caused by pericyte loss, suggesting other pericyte derived factors are important (41). Our recent work has also shown interactions between endothelial insulin receptors and VEGF signaling in endothelial cells during angiogenesis, illustrating a broader role of insulin signaling in vascular remodeling (42). Hence, insulin signaling appears to play a complex role in angiogenesis, and further work will be required to address the uncertainties described above.

### Diabetic Microvascular Disease and Pericytes

Diabetes dysregulates many essential endothelial–pericyte signaling mechanisms (39, 43-46). Moreover, there is good evidence that diabetes reduces the abundance and alters the function of retinal pericytes; this is thought to promote diabetic retinopathy, characterized by early vasoregression, followed by exaggerated regeneration of friable leaky neovessels (39, 44). Importantly, venous dilatation is a defining characteristic of early diabetic retinopathy that correlates with microvascular disease in other organs (7, 47), and is even apparent in prediabetes (6). While the impact in other vascular beds is not well studied (5), electron microscopy of human skeletal muscle suggests diabetes produces similar attrition (46, 48), but the consequences are unknown. Notably, diabetic retinopathy is phenocopied by retinal ANGPT2 overexpression (38), whilst ANGPT1 overexpression can retard the development of diabetic retinopathy and nephropathy (49, 50). It has also been reported that topical application of insulin aids diabetic wound healing and neovascular maturation in an ANGPT1-dependent manner (51). Hence, pericyte dysfunction and altered angiopoietin biology are key drivers of diabetic microvascular disease. However, it remains unclear whether insulin receptor expression or downstream signaling is altered in pericytes in the context of diabetes, and this warrants careful exploration in future studies.

### Limitations

While our work has important strengths, described above, it is important to discuss some limitations. First, we used a Pdgfrb-Cre that is active in the germline, which allowed us to ensure loss of mural cell insulin receptors during immediate postnatal vascular development; however, this means that cells expressing *Pdgfrb* during embryogenesis are likely to have undergone insulin receptor deletion. Although we saw no differences in whole retina or lung insulin receptor protein, nor developmental or growth defects, we cannot discount the contribution of mural cell independent effects in vivo. However, our careful analysis of spatial Cre activity in the retina, analysis of isolated murine pericytes, and corroboration in human pericytes suggest that our pericyte observations are genuine. It is important to note that our analysis of Cre activity also indicates recombination in mural vascular smooth muscle cells lining arteries, which are very closely related to pericytes. Notably, published data have shown that insulin receptor knockout specifically in vascular smooth muscle cells protects against pathological intimal hyperplasia after arterial injury, supporting a broader role of insulin signaling in all vascular mural cells (52). Second, we have only explored developmental angiogenesis, and this may not be relevant to pathological states, so it will be important for future studies to explore this and also assess the vasculature of adult PIRKO mice, both in the quiescent state and during pathological angiogenesis. This will be important to define the wider relevance of pericyte insulin resistance to diabetic microvascular complications. Future studies should also explore extraretinal development during embryogenesis of PIRKO mice to understand the wider developmental implications. Next, while we observed robust mRNA knockdown of the insulin receptor in PIRKO pericytes, there was some residual (probably truncated) insulin receptor protein expression and we have not explored downstream insulin signaling pathways in pericytes to characterize residual signaling. Finally, although we explored the fundamental role of insulin receptors in murine and human pericytes, it is important not to assume these data describe the impact on pericyte insulin signaling in murine or human diabetes; indeed, diabetes induces insulin-signaling defects both at the receptor and multiple downstream nodes (8).

### Conclusions

We provide the first ever in vivo assessment of mice with pericyte insulin receptor knockout and demonstrate impaired vascular quiescence and venous patterning during developmental retinal angiogenesis. We implicate diminished secretion of ANGPT1 by pericytes, resulting in impaired venous plexus endothelial cell Tie2 signaling to the transcription factor FoxO1, leading to augmented ANGPT2 expression. This vascular and molecular phenotype has clear parallels with diabetic retinopathy and future studies should explore the role of diminished pericyte insulin signaling in this disease.

## Data Availability

All datasets generated during and/or analyzed during the current study are not publicly available but are available from the corresponding author on reasonable request.

## Abbreviations

ANGPT2: angiopoietin-2
ANOVA: analysis of variance
BSA: bovine serum albumin
HBPC: human brain pericyte
HUVEC: human umbilical vein endothelial cell
PCR: polymerase chain reaction
PDGF-B: platelet derived growth factor-β
PIRKO: pericyte insulin receptor knockout mice
SEM: standard error of the mean
VEGF: vascular endothelial growth factor

## Additional Information

**Disclosures:** None.

## References

[1] Armulik A , Genové G, Betsholtz C. Pericytes: developmental, physiological, and pathological perspectives, problems, and promises. Dev Cell. 2011;21(2):193-215. 21839917 10.1016/j.devcel.2011.07.001

[2] Potente M , Gerhardt H, Carmeliet P. Basic and therapeutic aspects of angiogenesis. Cell. 2011;146(6):873-887. 21925313 10.1016/j.cell.2011.08.039

[3] Lindahl P , Johansson BR, Levéen P, Betsholtz C. Pericyte loss and microaneurysm formation in PDGF-B-deficient mice. Science. 1997;277(5323):242-245. 9211853 10.1126/science.277.5323.242

[4] Mäe MA , He L, Nordling S, et al. Single-cell analysis of blood-brain barrier response to pericyte loss. Circ Res. 2021;128(4):e46-e62. 33375813 10.1161/CIRCRESAHA.120.317473 PMC10858745

[5] Warmke N , Griffin KJ, Cubbon RM. Pericytes in diabetes-associated vascular disease. J Diabetes Complications. 2016;30(8):1643-1650. 27592245 10.1016/j.jdiacomp.2016.08.005

[6] Nguyen TT , Wang JJ, Wong TY. Retinal vascular changes in pre-diabetes and prehypertension: new findings and their research and clinical implications. Diabetes Care. 2007;30(10):2708-2715. 17595350 10.2337/dc07-0732

[7] Klein R , Myers CE, Lee KE, Gangnon R, Klein BE. Changes in retinal vessel diameter and incidence and progression of diabetic retinopathy. JAMA Ophthalmol. 2012;130(6):749-755. 10.1001/archophthalmol.2011.2560 PMC3357449 22332203

[8] Cubbon RM , Ali N, Sengupta A, Kearney MT. Insulin- and growth factor-resistance impairs vascular regeneration in diabetes mellitus. Curr Vasc Pharmacol. 2012;10(3):271-284. 22239629 10.2174/157016112799959305

[9] Vanlandewijck M , He L, Mäe MA, et al. A molecular atlas of cell types and zonation in the brain vasculature. Nature. 2018;554(7693):475-480. 29443965 10.1038/nature25739

[10] King GL , Goodman AD, Buzney S, Moses A, Kahn CR. Receptors and growth-promoting effects of insulin and insulinlike growth factors on cells from bovine retinal capillaries and aorta. J Clin Invest. 1985;75(3):1028-1036. 2984251 10.1172/JCI111764 PMC423655

[11] Brüning JC , Michael MD, Winnay JN, et al. A muscle-specific insulin receptor knockout exhibits features of the metabolic syndrome of NIDDM without altering glucose tolerance. Mol Cell. 1998;2(5):559-569. 9844629 10.1016/s1097-2765(00)80155-0

[12] Foo SS , Turner CJ, Adams S, et al. Ephrin-B2 controls cell motility and adhesion during blood-vessel-wall assembly. Cell. 2006;124(1):161-173. 16413489 10.1016/j.cell.2005.10.034

[13] Muzumdar MD , Tasic B, Miyamichi K, Li L, Luo L. A global double-fluorescent Cre reporter mouse. Genesis. 2007;45(9):593-605. 17868096 10.1002/dvg.20335

[14] Pitulescu ME , Schmidt I, Benedito R, Adams RH. Inducible gene targeting in the neonatal vasculature and analysis of retinal angiogenesis in mice. Nat Protoc. 2010;5(9):1518-1534. 20725067 10.1038/nprot.2010.113

[15] RRID:AB_ 11213678. http://antibodyregistry.org/AB_11213678.

[16] RRID:AB_2082660. http://antibodyregistry.org/AB_2082660.

[17] RRID:AB_2106495. http://antibodyregistry.org/AB_2106495.

[18] RRID:AB_354770. http://antibodyregistry.org/AB_354770.

[19] RRID:AB_2100105. http://antibodyregistry.org/AB_2100105.

[20] RRID:AB_2893028. http://antibodyregistry.org/AB_2893028.

[21] RRID:AB_884568. http://antibodyregistry.org/AB_884568.

[22] RRID:AB_2536183. http://antibodyregistry.org/AB_2536183.

[23] RRID:AB_2534104. http://antibodyregistry.org/AB_2534104.

[24] Tigges U , Welser-Alves JV, Boroujerdi A, Milner R. A novel and simple method for culturing pericytes from mouse brain. Microvasc Res. 2012;84(1):74-80. 22484453 10.1016/j.mvr.2012.03.008 PMC3748138

[25] RRID:AB_2212507. http://antibodyregistry.org/AB_2212507.

[26] RRID:AB_2893027. http://antibodyregistry.org/AB_2893027.

[27] RRID:AB_675659. http://antibodyregistry.org/AB_675659.

[28] RRID:AB_2714189. http://antibodyregistry.org/AB_2714189.

[29] RRID:AB_2280448. http://antibodyregistry.org/AB_2280448.

[30] RRID:AB_772210. http://antibodyregistry.org/AB_772210.

[31] RRID:AB_772206. http://antibodyregistry.org/AB_772206.

[32] Daly C , Wong V, Burova E, et al. Angiopoietin-1 modulates endothelial cell function and gene expression via the transcription factor FKHR (FOXO1). Genes Dev. 2004;18(9):1060-1071. 15132996 10.1101/gad.1189704 PMC406295

[33] Chu M , Li T, Shen B, et al. Angiopoietin receptor Tie2 is required for vein specification and maintenance via regulating COUP-TFII. Elife. 2016;5:e21032. 28005008 10.7554/eLife.21032 PMC5218530

[34] Wilhelm K , Happel K, Eelen G, et al. FOXO1 couples metabolic activity and growth state in the vascular endothelium. Nature. 2016;529(7585):216-220. 26735015 10.1038/nature16498 PMC5380221

[35] Riddell M , Nakayama A, Hikita T, et al. aPKC controls endothelial growth by modulating c-Myc via FoxO1 DNA-binding ability. Nat Commun. 2018;9(1):5357. 30559384 10.1038/s41467-018-07739-0 PMC6297234

[36] Ren B , Deng Y, Mukhopadhyay A, et al. ERK1/2-Akt1 crosstalk regulates arteriogenesis in mice and zebrafish. J Clin Invest. 2010;120(4):1217-1228. 20237411 10.1172/JCI39837 PMC2846043

[37] Gaengel K , Genové G, Armulik A, Betsholtz C. Endothelial-mural cell signaling in vascular development and angiogenesis. Arterioscler Thromb Vasc Biol. 2009;29(5):630-638. 19164813 10.1161/ATVBAHA.107.161521

[38] Pfister F , Wang Y, Schreiter K, et al. Retinal overexpression of angiopoietin-2 mimics diabetic retinopathy and enhances vascular damages in hyperglycemia. Acta Diabetol. 2010;47(1):59-64. 19238311 10.1007/s00592-009-0099-2

[39] Park SW , Yun JH, Kim JH, Kim KW, Cho CH, Kim JH. Angiopoietin 2 induces pericyte apoptosis via α3β1 integrin signaling in diabetic retinopathy. Diabetes. 2014;63(9):3057-3068. 24722242 10.2337/db13-1942

[40] Teichert M , Milde L, Holm A, et al. Pericyte-expressed Tie2 controls angiogenesis and vessel maturation. Nat Commun. 2017;8:16106. 28719590 10.1038/ncomms16106 PMC5520106

[41] Park DY , Lee J, Kim J, et al. Plastic roles of pericytes in the blood-retinal barrier. Nat Commun. 2017;8:15296. 28508859 10.1038/ncomms15296 PMC5440855

[42] Walker AMN , Warmke N, Mercer B, et al. Endothelial insulin receptors promote VEGF-a signaling via ERK1/2 and sprouting angiogenesis. Endocrinology. 2021;162(8):bqab104. 34037749 10.1210/endocr/bqab104 PMC8223729

[43] Hammes HP , Lin J, Renner O, et al. Pericytes and the pathogenesis of diabetic retinopathy. Diabetes. 2002;51(10):3107-3112. 12351455 10.2337/diabetes.51.10.3107

[44] Hammes HP , Lin J, Wagner P, et al. Angiopoietin-2 causes pericyte dropout in the normal retina: evidence for involvement in diabetic retinopathy. Diabetes. 2004;53(4):1104-1110. 15047628 10.2337/diabetes.53.4.1104

[45] Tanii M , Yonemitsu Y, Fujii T, et al. Diabetic microangiopathy in ischemic limb is a disease of disturbance of the platelet-derived growth factor-BB/protein kinase C axis but not of impaired expression of angiogenic factors. Circ Res. 2006;98(1):55-62. 16306442 10.1161/01.RES.0000197842.38758.45

[46] Hayden MR , Yang Y, Habibi J, Bagree SV, Sowers JR. Pericytopathy: oxidative stress and impaired cellular longevity in the pancreas and skeletal muscle in metabolic syndrome and type 2 diabetes. Oxid Med Cell Longev. 2010;3(5):290-303. 21150342 10.4161/oxim.3.5.13653 PMC3154033

[47] Wong TY , Shankar A, Klein R, Klein BE. Retinal vessel diameters and the incidence of gross proteinuria and renal insufficiency in people with type 1 diabetes. Diabetes. 2004;53(1):179-184. 14693713 10.2337/diabetes.53.1.179

[48] Tilton RG , Faller AM, Burkhardt JK, Hoffmann PL, Kilo C, Williamson JR. Pericyte degeneration and acellular capillaries are increased in the feet of human diabetic patients. Diabetologia. 1985;28(12):895-900. 4092858 10.1007/BF00703132

[49] Jeansson M , Gawlik A, Anderson G, et al. Angiopoietin-1 is essential in mouse vasculature during development and in response to injury. J Clin Invest. 2011;121(11):2705-2710. 10.1172/JCI46322 PMC3104773 21606590

[50] Cahoon JM , Rai RR, Carroll LS, et al. Intravitreal AAV2.COMP-Ang1 prevents neurovascular degeneration in a murine model of diabetic retinopathy. Diabetes. 2015;64(12):4247-4259. 26340930 10.2337/db14-1030 PMC4657578

[51] Li C , Yu T, Liu Y, Chen X, Zhang X. Topical application of insulin accelerates vessel maturation of wounds by regulating angiopoietin-1 in diabetic mice. Int J Low Extrem Wounds. 2015;14(4):353-364. 26349856 10.1177/1534734615600590

[52] Li Q , Fu J, Xia Y, et al. Homozygous receptors for insulin and not IGF-1 accelerate intimal hyperplasia in insulin resistance and diabetes. Nat Commun. 2019;10(1):4427. 31562314 10.1038/s41467-019-12368-2 PMC6765023

